# Associations of perceived neighbourhood safety from traffic and crime with overweight/obesity among South African adults of low-socioeconomic status

**DOI:** 10.1371/journal.pone.0206408

**Published:** 2018-10-31

**Authors:** Pasmore Malambo, Anniza De Villiers, Estelle V. Lambert, Thandi Puoane, Andre P. Kengne

**Affiliations:** 1 Faculty of Community and Health Sciences School of Public Health, University of Western Cape, Cape Town, South Africa; 2 Non-communicable Diseases Unit, South African Medical Research Council, Cape Town, South Africa; 3 Division of Exercise Science and Sports Medicine, Department of Human Biology Faculty of Health Sciences, University of Cape Town, Cape Town, South Africa; TNO, NETHERLANDS

## Abstract

**Background:**

The relationship between perceived neighbourhood safety from traffic and crime with overweight/obesity can provide intervention modalities for obesity, yet no relevant study has been conducted in sub-Saharan African contexts. We investigated the association between perceived neighbourhood safety from traffic and crime with overweight/obesity among urban South African adults.

**Methods:**

This cross-sectional study included 354 adults aged ≥35 years drawn from the Prospective Urban Rural Epidemiology (PURE) cohort study. The Neighborhood Walkability Scale-Africa (NEWS-A) was used to evaluate the perceived neighbourhood safety. Adjusted odds ratios (ORs) and 95% confidence intervals (CIs) were calculated to examine the associations between perceived neighborhood safety and overweight/obesity defined “normal weight” and “overweight/obese” using the 25 Kg/m^2^ cutoff criterion.

**Results:**

In the overall sample, adults who agreed that “the speed of traffic on most nearby roads in their neighborhood was usually slow” were less likely to be overweight/obese (adjusted OR = 0.42; 95%CI 0.23–0.76). Those who agreed that “there was too much crime in their neighborhood to go outside for walks or play during the day” were more likely to be overweight/obese (OR = 2.41; 1.09–5.29). These associations were driven by significant associations in women, and no association in men, with significant statistical interactions.

**Conclusion:**

Perceived neighborhood safety from traffic and crime was associated with overweight/obesity among South African adults. Our findings provide preliminary evidence on the need to secure safer environments for walkability. Future work should also consider perceptions of the neighbourhood related to food choice.

## Background

Overweight and obesity are major health challenges in both low- and high-income countries [1]. The epidemic of overweight and obesity has been associated with non-communicable diseases (NCDs) [2], and increased health care costs [3] including in South Africa [4,5]. NCDs are predicted to account for seven out of every ten deaths in developing countries [6]. Similarly, it is estimated that at least 2.8 million people die each year as a result of overweight or obesity globally [7]. Currently, South Africa is undergoing a rapid epidemiological transition and has the highest prevalence of obesity in sub-Saharan Africa (SSA) [8]. Compared to high-income countries, obesity trends in South Africa have shown an increase between 2003 and 2012, with a prevalence in women between the ages of 45–54 years increasing from 40% to more than 55% [9,10]. This trend has been facilitated by high rate of urbanization, stable socioeconomic status and Western lifestyle that majority of South Africans have adopted [11]. While the causes of overweight and obesity are multifaceted, new ideas have emerged on the relationship between environment and weight status [12]. The environment is increasingly being implicated as an important modulator of energy intake (food choice) and expenditure (physical activity), and is considered to be a driving force behind the increased trends of overweight/obesity across all age groups [13,14]. For example, dietary habits and physical activity were significantly associated with weight status among college students [15]. In the South African context, factors such as high levels of traffic incidents [16], crime rates [17], westernized lifestyles [8], low levels of physical activity [18] and sedentary behaviors [19] have been reported. Previously, public health prevention efforts to combat obesity have largely focused on identifying and reducing individual level risk factors, particularly physical inactivity and poor eating habits, with little effect. Thus, ecological models of health behavior have been proposed as important conceptual frameworks to address the multiple correlates of obesity [20]. According to Assari, Caldwell and Zimmerman [21], neighborhood dissatisfaction has been shown to be associated with an increased prevalence of chronic conditions. Equally, multi-level interventions have the potential to influence health behaviors among individuals [22]. Consequently, this has led to a growing recognition that strategies which target individual behaviors, in addition to the context within which such behaviors arise, have the greatest public health impact [23]. Furthermore, there has been an increase in research to understand the relationship between neighbourhood environment and obesity [24].

Perceived neighbourhood safety has been associated with overweight/obesity. For example, areas that were safe from traffic and crime [22], and where people knew and trusted each other, had a lower BMI [25,26]. In addition, perceptions of security and crime rates were associated with higher prevalence of overweight [26]. Even among Portuguese children, obesity was lower in safe environments where they could walk/cycle at night and during the day [27]. However, these studies have been conducted in high-income developed countries with different neighbourhood features from low-income countries [22]. Nevertheless, one cross-sectional study conducted in Nigeria revealed that unsafe environment from traffic and crime at night was associated with overweight [22]. Subsequently, Oyeyemi et al [22] advocated for more evidence-based African studies to be conducted on the relationship between neighbourhood environment and overweight/obesity and further to develop interventions that can accommodate the African context across all age groups. Therefore, the aim of the current study was to investigate the association between perceived neighbourhood safety from traffic and crime and overweight/obesity among urban South African adults.

## Methodology

### Study setting and population

The current study was conducted in Langa Township, which is one of the old suburbs in the City of Cape Town in the Western Cape Province. The township is located in the Cape Flats, 11 km south-east of the center of Cape Town. The township encloses 3.09km^2^ of the total land area, with an estimated population density of 17,000/km^2^ [28]. The Township is predominantly black (99.1%) with equal sex distribution. About 40% of the population completed grade 12 or a higher education, 60% are employed and 72% earn less than R3, 200 ($1 ≈ ZAR14.00) per month [28]. In 2016, the provincial community-reported serious crime was estimated at 24.2% [29] with pedestrian fatalities of less than 10% in Langa Township [30].

Participants in the current study were drawn from the Langa Township site of the Prospective Urban Rural Epidemiology (PURE) cohort study in Cape Town, South Africa. The cohort, established in 2009, included 2064 Black South African men and women aged 35–70 years. Included participants had to: (1) be aged 35–70 years, (2) be living within an identified household, and (3) have no disability that precluded walking.

### Study design and sample size

This was a cross-sectional survey nested within PURE study. The data were collected from 2014 to 2015. A total sample of 354 participants, both men and women, who provided complete data on anthropometric measurements, participated in the study.

### Data collection procedure

The data collection followed baseline procedures developed in 2009 [31]. Participants were interviewed in the language of their choice. Participants were invited to a convenient center (the community school premises or churches), where trained research assistants carried out all physical measurements. Structured, socio-demographic and lifestyle questionnaires were used. NEWS-Africa questionnaire was used to assess perceived neighbourhood safety [32].

### Socio-demographic characteristics

Socio-demographic characteristics included age, gender, marital status, level of education, employment status, family income, car use, smoking and alcohol use. Participants’ age was grouped into four categories: 35 to 44, 45 to 54, 55 to 64, and over 65 years. Marital status was classified as single, married and widowed/divorced. Education level was classified as primary, secondary and tertiary education. Employment status was classified as unemployed and employed, income levels were categorized as less than R 2000 and more than R 2000 (R 2000 ≈ $157) and car use was defined as less than once a week and a few times a week. Both smoking and alcohol use were defined as “no” and “yes”.

### Anthropometric measures

Body height and weight were measured following standard anthropometric methods. Height was measured to the nearest 0.1 cm in bare feet, with participants standing upright, using a portable tape measure. Weight was measured to the nearest kilogram, with participants lightly dressed, using a portable bathroom weighing scale calibrated (Soehnle, Germany). Body mass index (BMI) was calculated as weight (kg) divided by the square of the height (m^2^). The WHO principal cutoff points for BMI [2] were used to create the categories: underweight (<18.5 kg/m^2^), normal weight (18.5- < 25 kg/m^2^), overweight (25-< 30 kg/m^2^), and obese (≥ 30 kg/m^2^). In the current study, participants were categorized based on their BMI as “normal weight” and “overweight/obese” using the 25 Kg/m^2^ cutoff criterion.

### Neighborhood safety assessment

An adapted self-administered version of the Neighborhood Environment Scale-Africa (NEWS-A) was used to assess perception of neighborhood safety factors [32]. The original NEWS items [33] was expanded to 89 scores (76 individual NEWS items and 13 computed scales). Several modifications were made to individual items, and some new items were added to capture important attributes in the African environment. Overall, 95% of all NEWS-Africa scores (items plus computed scales) demonstrated evidence of “excellent” (Intra-class correlation coefficients > .75%) or “good” (Intra-class correlation coefficients = 0.60 to 0.74) reliability. Seven (53.8%) of the 13 computed NEWS scales demonstrated “excellent” agreement and the other six had “good” agreement [32]. In the current study, the sub-scale on safety from traffic and crime were used.

Participants responded to the following items on perception of their safety from traffic: *There is so much traffic along nearby roads that it is difficult or unpleasant to walk or play in my neighborhood; The speed of traffic on most nearby roads in my neighborhood is usually slow; Most drivers exceed the speed limits (drive very fast) in my neighborhood; Walking or playing is dangerous in my neighborhood because of careless or aggressive driving; It could be dangerous to ride a bicycle in or near my neighborhood because of speed of traffic; I am worried about letting my child play or walk in my neighborhood and local streets because I am afraid of them being injured by a car)*; and for crime: *There is a lot of crime in my neighborhood; There is too much crime in my neighborhood to go outside for walks or play during the day; There is too much crime in my neighborhood to go outside for walks or play at night; There are groups of people or gangs (rascals*, *hooligans*, *thugs) in my neighborhood who make me feel threatened when I go out)*. Participants were instructed to consider neighborhood as the area within a 15–20 min walk from their home. These items were presented with the response options: strongly disagree, somewhat disagree, somewhat agree, strongly agree and not applicable in some items. All items except speed of traffic on nearby roads were reverse scored. For the purpose of data analysis, responses to each of the environmental variables were collapsed into categories of “agree” (strongly agree and somewhat agree) and “disagree” (strongly disagree and somewhat disagree). The test-retest of the mean score for both traffic and crime sub-scales were excellent (ICCs >.75%) [32].

### Ethical considerations

The study was conducted in accordance with the principles of the Helsinki declaration. All participants provided signed informed consent prior to participating in the study, which was approved by the Senate Higher Degrees committee and Research Committees of the University of the Western Cape, South Africa (Registration **#13/6/18**).

### Statistical analysis

Descriptive statistics were calculated for all variables. The independent association of perceived neighbourhood safety with overweight/obesity as the dependent variable was examined using logistic regression analysis. Adjusted odd ratios (OR) and 95% confidence interval (CI) were calculated for each neighborhood safety variable. Interactions between neighbourhood safety factors and gender for prevalent overweight/obesity risk were explored by including interaction terms in regression models containing the main effects of gender and the safety factor of interest.

## Results

Table 1 (S1 Dataset) shows that a total of 354 participants comprising 77.1% women and 22.9% men, with a mean age of 56.2 ± 10.6 years and mean BMI of 31.7 ± 9.7kg/m^2^, participated in this study. Compared with normal weight participants, overweight/obese adults (n = 251, 70.9% of the sample) were more likely to be older (38.6%), women (84.9%), unemployed (82.9%), non-smokers (45.4%) and 51.0% used alcohol (all p<0.05; Table 1). A marginally lower proportion of overweight/obese adults compared with their normal weight counterparts perceived that the speed of traffic in their neighbourhood was slow (52.6% vs 66.0%, p = 0.021). Similarly, 66.9% (n = 168) adults perceived that high crime during the day prevented them from walking or playing outside in their neighborhood compared to their 77.7% (n = 80) counterparts. The difference between BMI category was significant (p ≤ 0.05; S1 Table continues.)

**Table 1:** Socio-demographic characteristics of participants by weight status categories.

Characteristics	Body Mass Index			Population
	Normal weight	Overweight		
	N = 103 (29.1)	N = 251(70.9)	*p*-value	N = 354
	**Mean ± SD**	**Mean ± SD**		**Mean ± SD**
**Age (years)**	52.9 ±10.1	57.5 ± 10.5	**<0.001**†	56.2 ± 10.6
**BMI (kg/m**^**2**^**)**	21.4 ±2.4	35.9 ± 8.3	**<0.001**†	31.7 ± 9.7
	**N (%)**	**N (%)**		**N (%)**
**Age group**			**0.007**	
35–44	22 (21.4)	35 (13.9)		57 (16.1)
45–54	36 (35.0)	56 (22.3)		92 (26.0)
55–64	29 (28.2)	97 (38.6)		126 (35.6)
65 +	16 (15.5)	63 (25.1)		79 (22.3)
**Gender**			**< 0.001**††	
Male	43 (41.7)	38 (15.1)		81 (22.9)
Female	60 (58.3)	213 (84.9)		273 (77.1)
**Level of education**			0.652	
Primary	22 (21.4)	65 (25.9)		87 (24.6)
Secondary	68 (60.6)	158 (62.9)		226 (63.8)
Tertiary	13 (12.6)	28 (11.2)		41 (11.6)
**Employment status**			**0.032**††	
Unemployed	75 (72.8)	208 (82.9)		283 (79.9)
Employed	28 (27.2)	43 (17.1)		71 (20.1)
**Marriage status**			0.411	
Single	52 (50.5)	111 (44.2)		163 (46.0)
Married	28 (27.2)	86 (34.3)		114 (32.2)
Widowed/divorced	23 (22.3)	54 (21.5)		77 (21.8)
**Monthly income**			0.866	
≤ R 2000	18 (17.5)	42 (16.7)		294 (83.1)
≥ R 2000	85 (82.5)	209 (83.3)		60 (16.9)
**Car use**			0.811	
Less than once a week	65 (63.1)	155 (61.8)		220 (62.1)
A few times a week	38 (36.9)	96 (38.2)		134 (37.9)
**smoking**			**0.002**††	
No	38 (36.9)	137 (54.6)		175 (49.4)
Yes	65 (63.1)	114 (45.4)		179 (50.6)
**Alcohol**			**<0.001**††	
No	27 (26.2)	123 (49.0)		150 (42.4)
Yes	76 (73.8)	128 (51.0)		204 (57.6)
**BMI prevalence**			**<0.001**††	
Normal weight	103 (100)	-		103 (29.1)
Overweight	-	66 (26.3)		66 (18.6)
Obese	-	185 (73.7)		185 (52.3)
**Sub-scale of traffic and crime (M±SD**)				
Safety from traffic	1.12 ±0.24	1.21±0.26	0.477	1.21±0.26
Safety from crime	1.16±0.30	1.20±0.33	0.231	1.19±0.32

M-mean; SD- standard deviation; BMI- body mass index;

^†^ - p-value based on independent t-test statistic;

^††^ - p-value based on chi-squared statistic,

All bold entries are significant (p<0.05)

Table 2 shows odd ratios from univariable and adjusted regression models for the association of each perceived neighbourhood safety item with prevalent overweight/obesity. In univariable analysis, participants who agreed that the speed of traffic in their neighbourhood was usually slow were less likely to be overweight/obese (OR = 0.57; CI = 0.35–0.92). Participants who perceived high crime during the day prevented them from walking and playing outside were more likely to be overweight/obese (OR = 1.72; 1.01–2.93).

**Table 2:** Crude and adjusted odds ratios of perceived neighborhood safety with overweight/obese and interactions by sex.

Variables	Body Mass Index		
	Overweight (Overall sample)		
	Crude ORs	Adjusted ORs‡	Sex and Safety
	OR (95%-CI)	OR (95%-CI)	P-value
**Perceived neighborhoods safety (ref. disagree)**			
**Safety from Traffic**			
There is so much traffic along nearby roads that it is difficult or unpleasant to walk or play in my neighborhood	0.94 (0.54–1.64)	1.74 (0.83–3.64)	0.340
The speed of traffic on most nearby roads in my neighborhood is usually slow	0.57 (0.35–0.92)*	0.42**(0.23–0.76)	**0.038**
Most drivers exceed the speed limits (drive very fast) in my neighborhood	0.92 (0.45–1.77	1.94 (0.65–5.81)	0.319
Walking or playing is dangerous in my neighborhood because of careless or aggressive driving	1.11 (0.55–2.25)	0.48 (0.15–1.61)	0.930
It could be dangerous to ride on bicycle in or near my neighborhood because of speed of traffic	1.08 (0.62–1.89)	1.11 (0.48–2.54)	0.958
I am worried about letting my child play or walk in my neighborhood and local streets because I am afraid of them being injured by a car	1.12 (0.51–2.46)	0.82 (0.31–2.18)	0.945
**Safety from crime**			
There is a lot of crime in my neighborhood	1.28 (0.66–2.46)	0.94 (0.31–2.88)	0.373
There is too much crime in my neighborhood to go outside from walks or play during the day	1.72 (1.01–2.93)*	2.41*(1.09–5.29)	**<0.001**
There is too much crime in my neighborhood to go outside for walks or play at night	1.19 (0.59–2.40)	1.03 (0.32–3.31)	0.611
There are groups of people or gangs (rascals, hooligans, thugs) in my neighborhood who make me feel threatened when I go out	1.18 (0.62–2.24)	1.43 (0.51–4.03)	0.127
**Sub-scales of safety from traffic and crime**			
Safety from traffic	2.67(0.33–21.73)	1.48(0.15–14.28)	**<0.001**
Safety from crime	1.59(0.74–3.42)	2.13(0.91–4.98)	**<0.001**

^**‡**^Adjusted for age, gender, employment, smoking and alcohol use;

*p < 0.05;

** p < 0.001

In adjusted models (and relevant gender interaction terms in the overall sample, Table 2), perceived slow speed from traffic (OR = 0.41; 0.23–0.72) and high crime during the day (OR = 2.20; 1.04–4.64) remained significantly associated with overweight/obese. In both cases, there was evidence of significant statistical interaction by gender in the association (both interaction p≤0.038). Anlyses stratified by gender indicated that the observed associations in the total sample were driven by significant associations in women, but not in men. Odd ratios and 95%CI men and women were 0.22 (0.12–2.67) and 0.32 (0.14–0.75) for perceived slow speed from traffic, and 1.070.62 (0.47–32.72 and 8.05 (2.08–31.18) for high crime during day. Furthermore, at sub-scale level, a similar interaction pattern with gender was observed for safety from traffic 3.41(2.21–5.25) and safety from crime 3.30(2.13–5.10) respectively.

## Discussion

The major finding from the present study was that slow speed from traffic and high crime during the day were significantly associated with overweight/obesity. This was driven primarily by significant associations in women, but not in men; an observation to be interpreted in the context of the small number of men in the study. The observed finding is significant, especially in countries undergoing transition, where safety concerns are growing with related increased urbanization.

Our findings are similar to those from other studies that reported safety from traffic in pooled data from 12 countries [20], general neighborhood score on safety [34] such as heavy traffic [35], safety from traffic [20] and safety from crime [36] from high-income countries, low-income countries [37] and among children [38] to be associated with overweight/obesity. In addition, in the crude odds ratios, the present study noted that high crime during the day was associated with overweight/obesity. Other studies on crime have shown similar results [39, 40], but one study did not [41]. The measures of crime and traffic used in the current study may not be sufficiently valid or sensitive to perceptions of safety, inviting better measures [42]. However, safety from crime among adults [43] and children and adolescents [44,45] have been associated with overweight/obesity in the expected direction. Furthermore, traffic calming strategies have been associated with improvements in overall health and health related behaviours [46]. For instance, a study conducted among Dutch adults noted that a general feeling of safety was associated with lower obesity [35]. It can be deduced that female adults who perceived threat from traffic and crime would be less likely to engage in outdoor regular physical activity. For instance, fear for personal safety due to crime and traffic may reduce mobility among adult individuals [47]. A study in South Africa found that older adults were unable to walk while carrying packages at a pace that would allow them to cross at an intersection in the time taken between a change in traffic signals [47]. Thus, individuals who experience fear may not engage in regular physical activity and are more likely to engage in obesogenic sedentary behaviors and have poorer diet quality. The current study demonstrates the importance of future programs, policies and infrastructure changes aimed at lowering the pandemic of overweight/obesity by improving perceived neighbourhood safety from both traffic and crime.

## Limitations and strengths of the study

Our study has some limitations to be highlighted when interpreting the results. Our findings are based on cross-sectional data; thus, no causal relationships could be established. Our participants were recruited from a single geographical area, which limited the variability in traffic and crime safety measures. Although a valid and reliable instrument was used, data were collected via a self-administered questionnaire, and reporting biases may have occurred. In addition, this is the first study that has used NEWS-Africa in an African context. Therefore, these results cannot be generalized to the South African urban adult population. Moreover, our sample was largely women recruited from one urban city, which could influence the effect in the logistic regression analysis. Additionally, the current study used perceived, as opposed to objective, measures of neighbourhood environments. Prior studies [48,49] indicate a poor level of agreement between objective and perceived measures of the built environment, yet it is not known which is more important to overweight/obesity [37]. Finally, our study did not include measures of the food environment, which may be important in explaining overweight/obesity. Despite these limitations, our findings strengthen the evidence base demonstrating that perceived neighbourhood safety can support people in meeting moderate physical activity recommendations, which in turn, aids in lowering the prevalence of overweight/obesity. Our study also contributes to the limited body of existing literature regarding perceived neighbourhood factors and overweight/obesity. We found that perceived environmental factors of low traffic speed, parents worried that their children may be injured by a car and high crime during the day were correlated with overweight/obesity in Langa Township in expected directions.

## Conclusion

Our results were consistent with previous research suggesting that perceived neighbourhood safety is related to overweight/obesity in the sub-Saharan African context. More importantly, our study suggests that perceptions of slow speed from traffic and high crime during the day are related to overweight/obesity. These associations were significant among women, because of low levels of outdoor physical activity such as walking for transport and leisure due to fear of being harmed. Future interventions are needed to provide safer environments by considering public health policies aimed at reducing traffic speed and volume, and improving safety [50] especially for women in sub-Saharan Africa.

## Supporting information

S1 Table (DOCX)

S1 Dataset (SAV)
