# Supplementary material for: Associations of perceived neighbourhood safety from traffic and crime with overweight/obesity among South African adults of low-socioeconomic status
Source: PLoS One. 2018 Oct 31;13(10):e0206408. doi: 10.1371/journal.pone.0206408 (PMC6209311; doi:10.1371/journal.pone.0206408)
Supplement: S1 Table — (DOCX) [file pone.0206408.s001.docx]

**Table 1 continues**

|  | **Body Mass Index** | | | |
| --- | --- | --- | --- | --- |
|  | **Normal weight** | **overweight/obese** |  | **Population** |
| **Characteristics** | **N=103 (29.1)** | **N=251 (70.9)** | **p-value** | **N=354** |
| **Safety from traffic and crime** |  |  |  |  |
| **Safety from traffic** |  |  |  |  |
| There is so much traffic along nearby roads that it is difficult or unpleasant to walk or play in my neighborhood | | | 1.826 |  |
| Agree | 81(78.6) | 200(79.7) |  | 281(79.4) |
| Disagree | 22(21.4) | 51(20.3) |  | 73(20.3) |
| The speed of traffic on most nearby roads in my neighborhood is usually slow | | | **0.021***††* |  |
| Agree | 68(66.0) | 132(52.6) |  | 200(56.5) |
| Disagree | 35(34.0) | 119(47.4) |  | 154(43.5) |
| Most drivers exceed the speed limits (drive very fast) in my neighborhood | | | 0.801 |  |
| Agree | 88(85.4) | 217(86.5) |  | 305(86.2) |
| Disagree | 15(14.6) | 34(13.5) |  | 49(13.8) |
| Walking or playing is dangerous in my neighborhood because of careless or aggressive driving | | | 0.776 |  |
| Agree | 91(88.3) | 219(87.3) |  | 310(87.6) |
| Disagree | 12(11.7) | 32(12.7) |  | 44(12.4) |
| It could be dangerous to ride on bicycle in or near my neighborhood because of speed of traffic |  |  | 0.782 |  |
| Agree | 81(78.6) | 194(77.3) |  | 275(77.7) |
| Disagree | 22(21.4) | 57(22.7) |  | 75(22.3) |
| I am worried about letting my child play or walk in my neighborhood and local streets because I am afraid of them being injured by a car | | | 0.778 |  |
| Agree | 93(90.3) | 229(91.2) |  | 322(91.0) |
| Disagree | 10(9.7) | 22(8.8) |  | 32(9.0) |
| **Safety from crime** |  |  |  |  |
| There is a lot of crime in my neighborhood | | | 462 |  |
| Agree | 89(86.4) | 209(83.3) |  | 298(84.2) |
| Disagree | 14(13.6) | 42(16.7) |  | 56(15.8) |
| There is too much crime in my neighborhood to go outside from walks or play during the day |  |  | **0.045***††* |  |
| Agree | 80(77.7) | 168(66.9) |  | 248(70.1) |
| Disagree | 23(22.3) | 83(33.1) |  | 106(29.9) |
| There is too much crime in my neighborhood to go outside for walks or play at night | | | 0.630 |  |
| Agree | 91(88.3) | 217(86.5) |  | 308(87.0) |
| Disagree | 12(11.7) | 34(13.5) |  | 46(13.0) |
| There are groups of people or gangs (rascals, hooligans, thugs) in my neighborhood who make me feel threatened when I go out | | | 0.614 |  |
| Agree | 88(85.4) | 209(83.3) |  | 297(83.9) |
| Disagree | 15(14.6) | 42(16.7) |  | 57(16.1) |

*†† - p-value based on chi-squared statistic, all bold entries are significant (p<0.05)*
